# Slow Stapler Firing May Reduce Intraoperative Staple-Line Air Leakage During Pulmonary Resection: A Retrospective Propensity Score-Matched Study

**DOI:** 10.7759/cureus.109716

**Published:** 2026-05-27

**Authors:** Ryohei Miyazaki, Masaya Tamura, Naoki Furukawa, Takashi Sakai, Yujiro Bunno, Marino Yamamoto, Hironobu Okada

**Affiliations:** 1 Department of Thoracic Surgery, Kochi Medical School, Kochi University, Nankoku, JPN

**Keywords:** air leakage, propensity score matching (psm), pulmonary resection, staple line, stapler firing speed

## Abstract

Background

Air leakage after pulmonary resection is one of the most common complications in thoracic surgery and is associated with prolonged chest drainage and increased healthcare costs. Although linear staplers are widely used for pulmonary parenchymal division, the association between stapler firing speed and intraoperative air leakage during pulmonary parenchymal division remains unclear.

Methods

This retrospective single-center study included 232 patients who underwent pulmonary resection for non-small cell lung cancer between October 2023 and December 2025.

Stapling procedures performed using conventional firing modes (approximately 6-10 seconds) were categorized as the fast mode, whereas procedures performed using the slow firing mode (≥20 seconds) were categorized as the slow mode. The primary endpoint was the presence of intraoperative air leakage originating from the staple line during the routine leak test. Surgical videos were retrospectively reviewed to identify staple-line-related air leaks. To adjust for baseline differences between groups, propensity score matching was performed using age, sex, Brinkman index, Goddard score, surgical approach, type of resection, reinforcement material use, and the number of stapler cartridges.

Results

Among the 232 patients, 147 were assigned to the fast mode group and 85 to the slow mode group. After propensity score matching, 83 matched pairs were analyzed. The incidence of staple-line air leak was lower in the slow mode group than in the fast mode group (n=9 (10.8%) vs. n=19 (22.9%); p=0.036). In univariable analysis, smoking history, Goddard score, and stapling speed were associated with air leakage. In multivariable logistic regression analysis, fast stapler firing (reference: slow firing) was significantly associated with intraoperative staple-line air leakage (OR 3.19; 95% CI: 1.1-6.2; p=0.03).

Conclusions

Although limited by device heterogeneity and its retrospective design, this study suggests that slower stapler firing may be associated with reduced intraoperative staple-line air leakage. These findings should be considered hypothesis-generating because complete separation of firing-speed effects from device-related factors was not feasible.

## Introduction

Linear staplers are widely used for pulmonary parenchymal division, and recent advances in stapling devices have substantially improved their safety and reliability. Nevertheless, intraoperative air leakage from the staple line is still encountered in clinical practice. Such leakage may occur due to tissue fragility, uneven compression, or suboptimal staple formation. Previous experimental studies have suggested that mechanical factors during stapling, including tissue compression and firing speed, may influence staple formation and tissue injury. In particular, slower firing and adequate tissue compression have been reported to promote more uniform staple formation and reduce tissue damage.

In recent years, several clinical studies in thoracic surgery have examined the impact of stapler firing speed on surgical outcomes. For example, slow firing during pulmonary artery division has been reported to reduce bleeding and oozing from the vascular stump [1,2]. These findings suggest that stapler firing speed may affect the mechanical stability of the staple line. However, most previous investigations have focused on vascular stapling and bleeding-related outcomes. In contrast, the potential influence of stapler firing speed on air leakage during pulmonary parenchymal division has not been adequately investigated. Moreover, many previous studies have mainly evaluated postoperative outcomes such as chest tube duration or prolonged air leak. However, these outcomes are influenced by multiple perioperative factors and may not directly reflect the mechanical integrity of the staple line itself.

Therefore, the clinical significance of stapler firing speed in preventing air leakage from lung parenchyma remains unclear. In the present study, we retrospectively analyzed patients undergoing pulmonary resection and evaluated intraoperative staple-line air leakage through surgical video review. To minimize potential confounding, propensity score matching was used to balance baseline characteristics between the fast and slow firing groups. By directly assessing air leakage during intraoperative leak tests, this study aimed to evaluate the association between stapler firing speed and intraoperative staple-line air leakage during pulmonary resection. To our knowledge, few clinical studies have directly evaluated the relationship between stapler firing speed and intraoperative staple-line air leakage during pulmonary parenchymal division.

The present study aimed to directly evaluate the association between stapler firing speed and intraoperative staple-line air leakage during pulmonary parenchymal division.

## Materials and methods

Study design and patient selection

This study was a single-center retrospective analysis conducted at Kochi Medical School Hospital, Nankoku, Japan. The study protocol was approved by the Ethical Review Board of Kochi University (approval number: 2025-91) prior to study initiation. The requirement for informed consent was waived due to the retrospective nature of the study. This study was reported in accordance with the Strengthening the Reporting of Observational Studies in Epidemiology (STROBE) reporting guideline.

A total of 238 patients who underwent curative pulmonary resection for non-small cell lung cancer at the Department of General Thoracic Surgery of Kochi University between October 2023 and December 2025 were initially reviewed. Patients with a history of prior thoracic surgery, those who received preoperative induction therapy, those with complete pleural adhesions, and those with insufficient preoperative or intraoperative data were excluded. Ultimately, 232 patients were enrolled in the study.

All enrolled cases underwent pulmonary resection using one of the four types of surgical staplers. The choice of stapler platform and firing mode was primarily based on surgeon preference and device availability during the study period. Because slow firing mode was commercially available only in the Signia™ stapling system (Medtronic, Minneapolis, Minnesota, United States) during the study period, complete separation of firing-mode effects from device-specific characteristics was not possible.

The primary endpoint was intraoperative staple-line air leakage identified during the routine leak test before chest closure. The duration of postoperative drain placement (>5 days) was set as a secondary endpoint. Clinical variables, including age, sex, and smoking history (Brinkman index), were collected. Smoking exposure was assessed using the Brinkman index, calculated as the number of cigarettes smoked per day multiplied by the duration of smoking in years. Higher Brinkman index values indicate greater cumulative smoking exposure and are associated with increased risk of smoking-related diseases. The Brinkman index was originally described by Brinkman and Coates [3]. As an assessment of the background lung condition, the low attenuation area (LAA) was measured, and the Goddard score was calculated. Emphysema severity was assessed using the Goddard scoring system on preoperative chest computed tomography (CT) images, as originally described by Goddard et al. [4]. Each lung was divided into three regions (upper, middle, and lower fields), resulting in six lung fields in total. Each field was visually scored according to the percentage of low-attenuation emphysematous area as follows: 0, no emphysema; 1, ≤25% involvement; 2, 26-50% involvement; 3, 51-75% involvement; and 4, >75% involvement. The scores from all six fields were summed to obtain a total Goddard score ranging from 0 to 24, with higher scores indicating more severe emphysema. Surgical variables included the surgical approach (thoracotomy, video-assisted thoracic surgery (VATS), or robot-assisted thoracic surgery (RATS)), the type of resection (wedge resection, segmentectomy, or lobectomy), stapler platform, and the use of staple-line reinforcement materials during stapling.

Staplers

Stapling speed was classified according to the preset firing mode and manufacturer specifications of each stapling system. Procedures completed within approximately 6-10 seconds were categorized as the fast mode, whereas those requiring approximately 20 seconds or longer were categorized as the slow mode.

The fast mode included the following devices: (1) powered ECHELON FLEX™ stapler (Ethicon, Johnson & Johnson, Cincinnati, Ohio, United States), (2) SureForm™ stapler (Intuitive Surgical, Sunnyvale, California, United States), and (3) the conventional normal mode of the Signia™ stapling system. In contrast, the slow mode consisted of (4) the manually activated slow firing mode of the Signia™ stapling system. Pulmonary parenchymal division was performed using one of the four surgical stapling devices.

**Figure 1:**
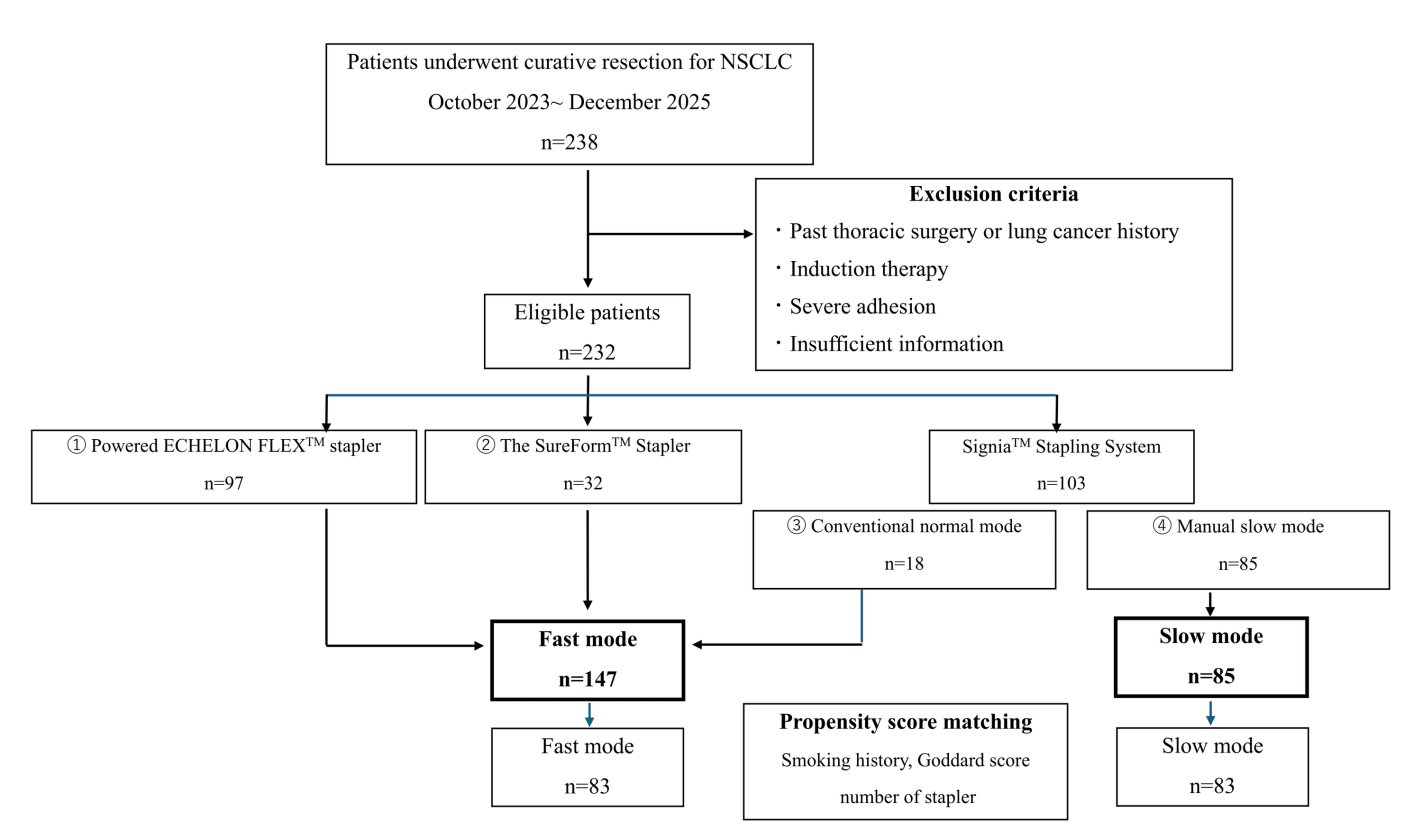
Flow diagram of patient selection A total of 238 patients who underwent pulmonary resection for NSCLC between October 2023 and December 2025 were screened. After exclusion criteria were applied, 232 patients were included in the analysis. Patients were categorized into fast and slow firing groups according to stapler firing time. Propensity score matching yielded 83 matched pairs for the final analysis. NSCLC: non-small cell lung cancer

A total of 147 patients were included in the fast mode group (1: n=97; 2: n=32; 3: n=18), while 85 patients were included in the slow mode group using device 4 (Figure 1). The primary endpoint, the presence of air leakage, was compared between the fast mode and slow mode groups. In addition, a supplementary analysis was performed using only cases in which the same device (Signia™ stapling system) was used, comparing the incidence of air leakage between the fast mode (n=18) and slow mode (n=85) groups. Because slow firing mode was available only in the Signia™ stapling system, a supplementary analysis restricted to cases using the same platform was conducted to minimize potential device-related bias.

Evaluation of intraoperative air leakage

The primary endpoint was the presence or absence of air leak from the staple line. Surgical videos of the enrolled cases were retrospectively reviewed. The presence of an air leak and its location were confirmed using the routine leak test performed before chest closure.

The leak test was routinely performed under saline immersion at an airway pressure of approximately 15-20 cmH₂O according to institutional practice. An air leak was considered present when air bubbles originated directly from the staple line or within approximately 5 mm of the staple line and was judged to be related to the stapling procedure. Air leaks occurring at sites unrelated to the stapling procedure or those considered to be caused by energy devices were excluded from the analysis. Two thoracic surgeons (RM and MT) independently reviewed the surgical videos. Discrepancies were resolved by consensus. Interobserver agreement for air leak detection, as assessed using Cohen's kappa coefficient, was excellent (k=0.82).

Statistics

Statistical analyses were performed using JMP Version 17.2.0 (SAS Institute, Cary, North Carolina, United States). Between-group comparisons were conducted using the Pearson χ² test or the Wilcoxon rank-sum test. A two-sided p-value of <0.05 was considered statistically significant. To adjust for differences in preoperative patient characteristics between the two groups, propensity score matching was applied. Propensity score matching was performed using 1:1 nearest-neighbor matching without replacement with a caliper width of 0.2 of the standard deviation of the logit of the propensity score. Covariate balance between groups after matching was assessed using standardized mean differences, with an absolute value greater than 0.1 considered to indicate a meaningful imbalance. Propensity score matching was performed using the following covariates: age, sex, Brinkman index, Goddard score, surgical approach, resection type, reinforcement material use, and the number of stapler cartridges. The stapler platform was not included in the propensity score model because of substantial collinearity with firing mode. Therefore, an additional sensitivity analysis restricted to cases using the same stapling platform (Signia™ stapling system) was performed to partially address potential device-related confounding.

After matching, comparisons between groups were performed using the McNemar test for categorical variables and the paired t-test or Wilcoxon signed-rank test for continuous variables. Covariate balance was assessed using the standardized difference, and an absolute standardized difference greater than 0.1 was considered to indicate a clinically meaningful imbalance. For the evaluation of predictive factors, multivariable logistic regression analysis was performed, and odds ratios (ORs) with 95% confidence intervals (CIs) were calculated.

## Results

Clinical characteristics

Patient characteristics, operative data, and the presence of air leak in the fast mode group (n=147) and slow mode group (n=85) are shown in Table 1. There were no significant differences between the two groups in terms of age or sex. Smoking history was numerically more frequent in the fast mode group, although the difference was not statistically significant.

**Table 1:** Baseline characteristics according to stapler firing mode Baseline characteristics were comparable between the two groups, with no significant differences in age or sex. Smoking history was more frequent in the fast firing group, although the difference did not reach statistical significance. Between-group comparisons were conducted using the Pearson χ² test or the Wilcoxon rank-sum test. VATS: video-assisted thoracic surgery; RATS: robot-assisted thoracic surgery

Variables	Fast mode	Slow mode	P-value (Wilcoxon rank-sum test)	P-value (χ² test)
	(n=147)	(n=85)		
Age (median (range))	74 (38-89)	74 (51-91)	0.52	-
Gender				
Male	78 (53.1%)	48 (56.5%)	-	0.28
Female	69 (46.9%)	37 (43.5%)		
Brinkman index (median (range))	320 (0-2700)	200 (0-2000)	0.17	-
Goddard score				
0-1	16 (10.9%)	18 (21.2%)	-	0.009
2-7	110 (74.8%)	57 (67%)		
8-12	21 (14.3%)	10 (11.8%)		
Number of staplers (median (range))	4 (1-8)	4 (1-7)	0.82	-
Reinforcement				
Present	6 (4.1%)	5 (2.3%)	-	0.32
None	141 (95.9%)	80 (97.7%)		
Approach				
Thoracotomy	6 (4.1%)	4 (4.7%)	-	0.81
VATS, RATS	141 (95.9%)	81 (95.3%)		
Procedure				
Wedge resection	32 (21.8%)	22 (25.9%)	-	0.77
Segmentectomy	64 (43.5%)	35 (41.2%)		
Lobectomy	51 (34.7%)	28 (32.9%)		
Air leakage (intraoperative)				
Present	35 (23.8%)	9 (10.6%)	-	0.011
None	112 (76.2%)	76 (89.4%)		
Persistent air leak (>5 days)				
Present	33 (22.4%)	16 (18.8%)	-	0.26
None	114 (77.6%)	69 (81.2%)		

The Goddard score was significantly higher in the fast mode group, indicating that this group included more patients with severe emphysema (p=0.009). The number of stapler cartridges used was numerically higher in the fast mode group. Regarding the surgical procedure, wedge resection tended to be more frequent in the slow mode group; however, these differences did not reach statistical significance.

Propensity score matching

Propensity score matching was performed using the following covariates: age, sex, Brinkman index, Goddard score, surgical approach, resection type, reinforcement material use, and the number of stapler cartridges used as covariates to balance baseline characteristics (Table 2). After matching, 83 pairs (83 vs. 83 cases) were included in the analysis. There was no significant difference in the duration of postoperative drain placement between the two groups. Comparison of the incidence of air leak between the two groups showed that the slow mode group (10.8%) had a significantly lower rate of air leak than the fast mode group (22.9%) (p=0.036) (Table 2).

**Table 2:** Baseline characteristics after propensity score matching After propensity score matching, the incidence of intraoperative staple-line air leakage was significantly lower in the slow firing group than in the fast firing group (10.8% vs. 22.9%; p=0.036). Between-group comparisons were conducted using the Pearson χ² test or the Wilcoxon rank-sum test. An asterisk (*) indicates a statistically significant difference. VATS: video-assisted thoracic surgery; RATS: robot-assisted thoracic surgery

Variables	Fast mode	Slow mode	P-value (Wilcoxon rank-sum test)	P-value (χ² test)
	(n=83)	(n=83)		
Age (median (range))	74 (38-89)	74 (51-91)	0.76	-
Gender				
Male	46 (55.4%)	48 (57.8%)	-	0.49
Female	37 (44.6%)	35 (42.2%)		
Brinkman index (median (range))	300 (0-2700)	240 (0-2000)	0.33	-
Goddard score				
0-1	15 (18%)	17 (20.5%)	-	0.67
2-7	58 (70%)	57 (68.7%)		
8-12	10 (12%)	9 (10.8%)		
Number of staplers (median (range))	4 (1-8)	4 (1-7)	0.91	-
Reinforcement				
Present	2 (2.4%)	3 (3.6%)		0.73
None	81 (97.6%)	80 (96.4%)		
Approach				
Thoracotomy	4 (4.8%)	4 (4.8%)	-	1
VATS, RATS	79 (95.2%)	79 (95.2%)		
Procedure				
Wedge resection	21 (25.2%)	22 (26.5%)	-	0.89
Segmentectomy	31 (37.4%)	33 (39.8%)		
Lobectomy	31 (37.4%)	28 (33.7%)		
Air leakage (intraoperative)				
Present	19 (22.9%)	9 (10.8%)	-	0.036*
None	64 (77.1%)	74 (89.2%)		
Persistent air leak (>5 days)				
Present	18 (21.7%)	16 (19.3%)	-	0.49
None	65 (78.3%)	67 (80.7%)		

Univariable and multivariable analysis for air leakage

The results of the univariable and multivariable analyses for the presence of air leak are shown in Table 3. In the univariable analysis, smoking history, Goddard score, and stapling speed were identified as significant factors. In the multivariable analysis, fast stapler firing (reference: slow firing) was independently associated with intraoperative staple-line air leakage (OR: 3.19; 95% CI: 1.1-6.2; p=0.03). The stapler platform was not a significant factor for the occurrence of air leakage (p=0.27). No statistically significant difference was observed among stapler platforms in the exploratory subgroup analysis (p=0.27) (Table 3).

**Table 3:** Univariable and multivariable logistic regression analyses for predictors of intraoperative staple-line air leakage In univariable analysis, smoking history, Goddard score, and stapler firing speed were significantly associated with intraoperative staple-line air leakage. Multivariable analysis identified fast stapler firing (reference: slow firing) as an independent predictor of air leakage (OR: 3.19; 95% CI: 1.1-6.2; p=0.03). The stapler platform was not significantly associated with air leakage occurrence (p=0.27). For the evaluation of predictive factors, multivariable logistic regression analysis was performed, and odds ratios (ORs) with 95% confidence intervals (CIs) were calculated. An asterisk (*) indicates a statistically significant difference.

Variable	Univariate analysis				Multivariate analysis			
	Odds ratio	95% CI		P-value	Odds ratio	95% CI		P-value
Gender (male vs. female)	1.4	0.79	2.53	0.24	-	-	-	-
Age (≥71 vs. <71)	1.02	0.97	1.03	0.76	-	-	-	-
Smoking history (≥600 vs. <600)	4.88	1.38	17.1	0.014	1.08	0.87	3.93	0.08
Goddard score (≥6 vs. <6)	3.46	1.03	13.2	0.04	1.18	0.99	1.28	0.07
Procedure (lobectomy vs. sub-lobar resection)	1.58	0.83	2.98	0.16	-	-	-	-
Reinforcement (present vs. absent)	1.23	0.34	3.68	0.73	-	-	-	-
Speed (fast vs. slow)	4.77	2.16	12.1	<0.001	4.22	2.1	7.6	0.003*
Stapler platform (except for Medtronic vs. Medtronic)	2.01	1.22	4.56	0.03	1.32	0.89	2.31	0.27
Number of staplers (≥4 vs. <4)	1.04	0.87	1.78	0.31	-	-	-	-

Sensitivity analysis

**Figure 2:**
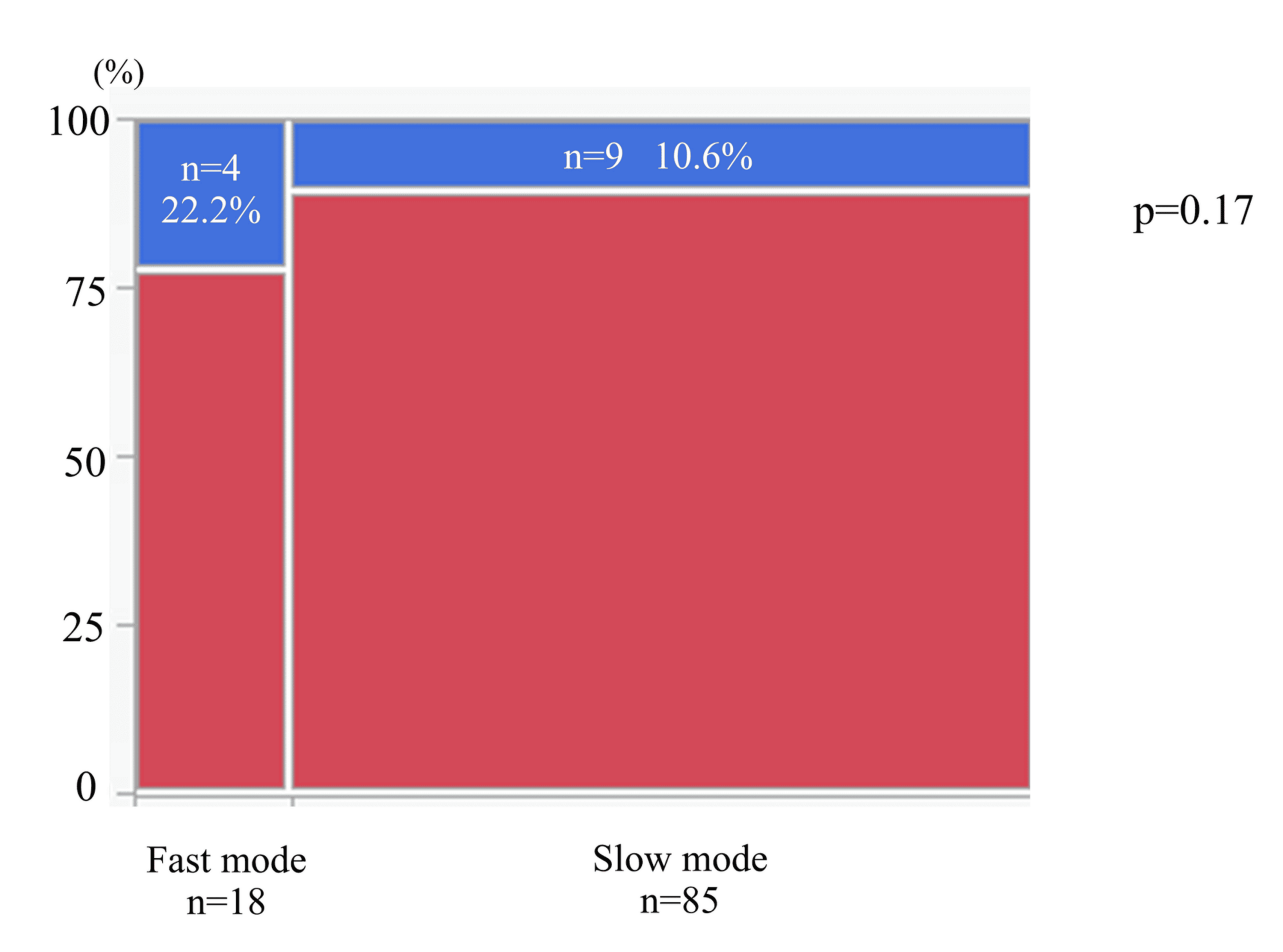
Comparison of air leakage rates between high-speed and low-speed modes of the Signia™ stapling system Air leakage rates were compared between the fast mode (n=18) and slow mode (n=85) groups. The blue area indicates cases with air leakage, whereas the red area indicates cases without air leakage. Although the incidence of air leakage was numerically higher in the fast mode group, the difference was not statistically significant (22.2% vs. 10.6%; p=0.17). Between-group comparisons were conducted using the Pearson χ² test.

**Figure 3:**
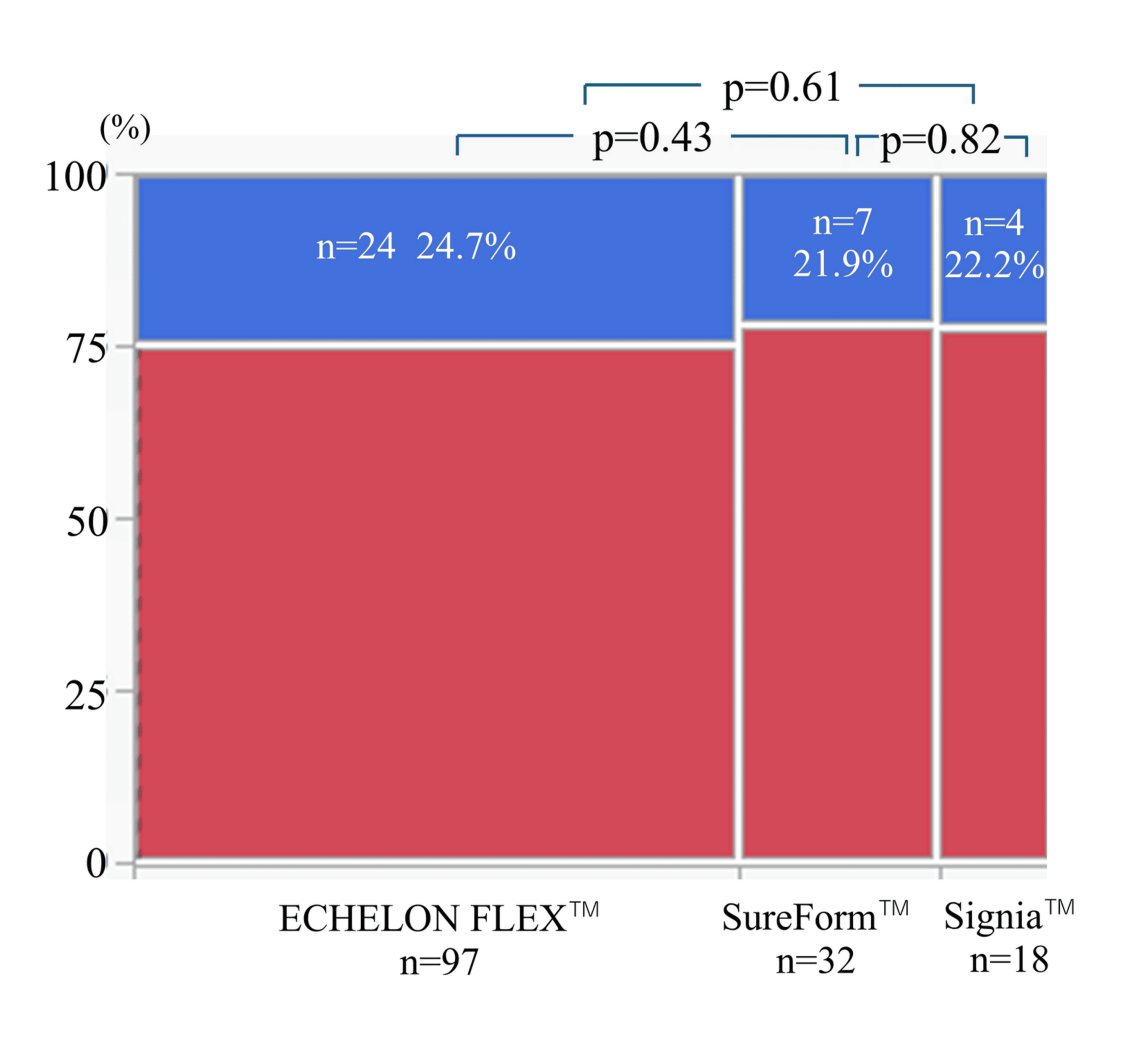
Comparison of air leakage rates among the three high-speed mode groups The blue area indicates cases with air leakage, whereas the red area indicates cases without air leakage. No significant differences in air leakage rates were observed among the three high-speed mode groups (24.7%, 21.9%, and 22.2%, respectively; p=0.43). Between-group comparisons were conducted using the Pearson χ² test.

In cases using the same device (Signia™ stapling system), air leakage was compared between the fast mode (n=18) and slow mode (n=85) groups. Although statistical significance was not reached because of the limited sample size, particularly in the fast Signia™ subgroup, the direction of the association was consistent with the primary analysis (22.2% vs. 10.6%; p=0.17) (Figure 2). To evaluate differences among device types, we compared the incidence of air leak among three fast mode groups: (1) the Powered ECHELON FLEX™ stapler (n=97), (2) the SureForm™ stapler (n=32), and (3) the conventional normal mode of the Signia™ stapling system (n=18) (Figure 3). No significant differences were observed among the groups (24.7%, 21.9%, and 22.2%, respectively; p=0.43).

## Discussion

In this retrospective study, slow stapler firing was associated with a lower incidence of intraoperative staple-line air leakage during pulmonary resection. This association remained significant after adjustment for measured confounding factors using propensity score matching and multivariable analysis. Although residual confounding, particularly related to the stapler platform, cannot be excluded, these findings suggest that stapler firing dynamics may influence staple-line integrity during pulmonary parenchymal division.

Air leak after pulmonary resection remains one of the most common complications in thoracic surgery, and prolonged air leak is known to be associated with prolonged chest tube duration and extended hospital stay [5,6]. Therefore, various preventive strategies, including staple-line reinforcement materials and sealants, have been proposed [7,8]. However, these approaches require additional devices and costs. In contrast, the findings of the present study suggest that optimizing a technical factor, namely, stapling speed, may reduce air leak without requiring additional materials, which may have important clinical implications. Because stapling speed can be modified without additional devices or costs, this technical adjustment may represent a readily implementable strategy for reducing staple-line air leakage in routine thoracic surgery. If validated prospectively, optimization of stapler firing dynamics may represent a simple and cost-free technical modification to improve staple-line integrity during pulmonary resection.

A notable strength of this study is that staple-line-related air leaks were directly evaluated through video analysis of intraoperative leak tests. In many previous studies, postoperative outcomes such as chest tube duration or the incidence of prolonged air leak have been used as endpoints [5,9]. However, these indicators may be influenced by multiple factors, including pleural adhesions, parenchymal fragility, and dissection techniques. In this study, we also examined the duration of postoperative drain placement; however, no difference was observed between the two groups. In the present study, we evaluated mainly air leaks originating from the staple line itself or from adjacent areas considered to be caused by the stapling procedure. This approach allowed a more direct assessment of the mechanical impact of stapling on lung tissue.

The mechanism by which slow firing reduces air leaks may be related to differences in tissue compression dynamics. Rapid firing may result in more abrupt tissue compression and localized mechanical stress within fragile lung parenchyma, whereas slower firing may allow more gradual tissue adaptation and more uniform staple formation. This hypothesis is supported by basic research in gastrointestinal surgery. For example, previous studies have reported that allowing sufficient pre-compression time after grasping tissue improves staple formation [10]. Because lung tissue is more fragile and contains more air than gastrointestinal tissue, the influence of compression dynamics may be even more pronounced.

In recent years, several studies in thoracic surgery have also suggested the potential benefits of slow firing. For instance, the use of slow firing mode during pulmonary artery division has been reported to reduce staple-line bleeding [1]. Another study demonstrated that slow firing significantly decreased persistent oozing from the vascular stump [2]. Although these studies focused on vascular structures, the present findings suggest that similar mechanisms may also apply to lung parenchyma. Indeed, among complications related to lung tissue stapling, air leak is reported to be the most frequent [11]. Therefore, the present findings, which focus on the technical factor of stapling speed, may contribute to improving the safety of staple lines during pulmonary resection.

Because firing mode was strongly linked to the stapler platform, separating the independent effects of firing speed from device-specific characteristics remains challenging. Although supplementary analyses demonstrated a consistent direction of association, residual device-related confounding cannot be excluded. Therefore, the present findings should be interpreted as hypothesis-generating, and prospective studies comparing firing speeds within the same stapling platform are warranted.

It should also be noted that the primary endpoint of this study was intraoperative staple-line leakage, rather than postoperative prolonged air leak. Although intraoperative leakage reflects the mechanical integrity of the staple line, prolonged air leak is influenced by multiple factors such as parenchymal fragility, fissure completeness, intraoperative manipulation, and postoperative lung expansion [5]. Therefore, it remains unclear whether the reduction in intraoperative leakage observed in this study would translate into a decrease in prolonged air leak. Prospective studies including postoperative outcomes will be required to clarify this issue.

This study has several limitations. First, residual confounding related to the stapler platform could not be completely excluded because the slow firing mode was available only in a single stapling system; the effect of firing speed could not be completely separated from device-specific characteristics. Second, this was a retrospective single-center study, and stapler selection depended on surgeon preference and device availability, which may have introduced selection bias. Third, the number of patients in some stapler subgroups, particularly the fast-firing Signia™ subgroup, was limited, reducing the statistical power of the sensitivity analysis. Fourth, actual intraoperative firing duration was not directly measured, and firing speed classification was based on preset device specifications. Finally, unmeasured factors, including cartridge selection, tissue thickness, and surgeon experience, may also have affected staple-line integrity. Future multicenter studies comparing different stapling systems are warranted.

## Conclusions

Slower stapler firing was associated with a lower incidence of intraoperative staple-line air leakage during pulmonary resection. Although the findings should be interpreted cautiously given the potential for device-related confounding, optimization of stapler firing dynamics may represent a potentially modifiable technical factor associated with staple-line integrity during pulmonary resection.
